# Primary lung cancer presenting with metastasis to the colon: a case report

**DOI:** 10.1186/1477-7819-10-127

**Published:** 2012-06-28

**Authors:** Hiroshi Sakai, Hiroyuki Egi, Takao Hinoi, Masakazu Tokunaga, Yasuo Kawaguchi, Manabu Shinomura, Tomohiro Adachi, Koji Arihiro, Hideki Ohdan

**Affiliations:** 1 Department of Gastroenterological Surgery, Hiroshima University Hospital, Hiroshima, Japan; 2 Department of Anatomical Pathology, Hiroshima University Hospital, 1-2-3 Kasumi Minami-ku, Hiroshima, 734-8551, Japan

**Keywords:** Colonic metastasis, Primary lung cancer, Squamous cell carcinoma

## Abstract

Although about 50% of lung cancers have distant metastasis at the time of initial diagnosis, colonic metastases are extremely rare. This report presents a rare clinical case of colonic metastasis from primary squamous cell carcinoma of the lung.

A 60-year-old female with anorexia and fatigue was referred to the department of pulmonary surgery in our hospital. The patient was diagnosed with primary squamous cell carcinoma of the lung, T2b N3 M1b Stage IV, and chemoradiotherapy was initiated. This treatment led to a good partial response in the primary lung lesion without any new metastatic lesions.

The patient developed left abdominal pain due to a bulky sigmoid colon tumor 6 months later, and was preoperatively diagnosed with primary colon cancer. She underwent colonic resection, and the pathology specimen demonstrated poorly differentiated squamous cell carcinoma that was suspected to be colonic metastasis from the primary lung cancer. The postoperative course was uneventful, and she was discharged. Chemotherapy for the lung cancer was scheduled in the department of pulmonary surgery.

This report presented a rare case of colonic metastasis from lung cancer. When patients with advanced primary lung cancer complain of abdominal symptoms, we should consider gastrointestinal tract metastasis from lung cancer.

## Background

Lung cancer is the most frequent cause of cancer death [1]. About 50% of all lung cancers have distant metastasis at the time of the initial diagnosis [2]. The brain, liver, adrenal glands, and bone are the most common sites of metastatic disease in patients with lung cancer [3]. Several autopsy studies reported that gastrointestinal metastasis from primary lung cancer occur in about 0.2 to 11.9% of cases [2,4-7]. A review of these studies indicates that the rate of metastasis of primary lung cancer to the gastrointestinal tract in autopsy studies is more common than originally thought. On the other hand, the clinical prevalence of symptomatic gastrointestinal metastasis of lung cancer is only 0.2 to 0.5% [5,8-11]. Within the gastrointestinal tract, the small bowel is the most common site of metastases from primary lung cancer [2]; however, the clinical prevalence of symptomatic colonic metastasis is extremely rare. This report presents a rare clinical case of colonic metastasis from primary squamous cell carcinoma of the lung.

## Case presentation

**Figure 1:**
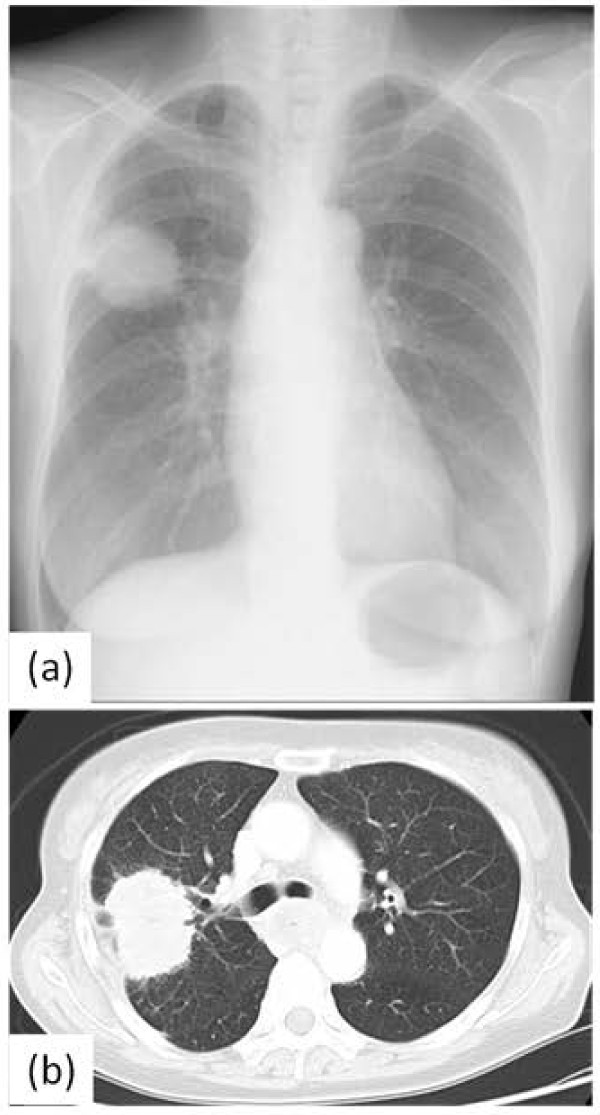
**Chest X-ray and computed tomography(CT) shows a huge tumor in the right lung field. (a)** Chest X-ray shows a 55 mm round mass in the right upper lung field. **(b**) Chest computed tomography scan reveals the mass in the right upper lobe with infiltration to B2 and B3a bronchus.

**Figure 2:**
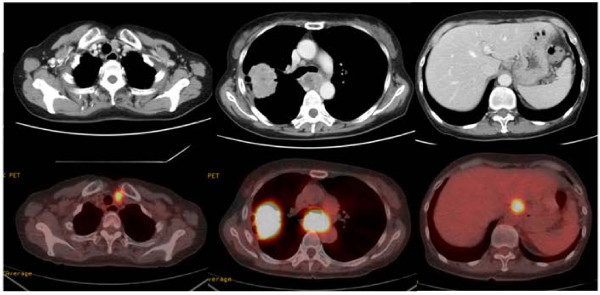
**Chest computed tomography (CT) scan.** The CT scan reveals the mass in the right upper lobe and enlarged lymph nodes of the upper mediastinum, around the upper thoracic esophagus and lesser curvature of the stomach. Positron emission tomography (PET)-CT reveals positive findings of the same lesions as the CT scan with no other positive lesion (maximum standardized uptake value: lung tumor 19.5, lymph nodes #2 L 9.3, #7 24.3, lesser curvature of the stomach 13.2).

A 60-year-old female with anorexia and fatigue was referred to the department of pulmonary surgery with a diagnosis of primary lung cancer. She had no past history of serious illnesses, operations or hospitalizations. The tumor markers were CEA 9.7 ng/ml, CYFRA 4.9 ng/ml, and SCC 0.6 ng/ml, respectively. A chest X-ray showed a 55 mm round mass in the right upper lung field (Figure 1a). Chest computed tomography (CT) revealed a mass in the right upper lobe with infiltration to the B2 and B3 bronchus and enlarged lymph nodes of the left upper mediastinum (#2 L), subcarina (#7) with infiltration to the esophagus and lesser curvature of the stomach (Figure 1b). In addition, positron emission tomography (PET)-CT revealed positive findings of the same lesions revealed by CT with no other positive lesion (maximum standardized uptake value (Max SUV): lung tumor 19.5, lymph nodes #2 L 9.3, #7 24.3, lesser curvature of the stomach 13.2) (Figure 2). A bronchoscopic biopsy specimen of B2 and B3 revealed squamous cell carcinoma. Upper gastrointestinal endoscopy showed an ulcerative lesion in the upper thoracic esophagus and a biopsy specimen from the lesion revealed invasion of the metastatic lymph nodes to the esophagus. The patient was diagnosed with primary squamous cell carcinoma of the lung, T2b N3 M1b (extrathoracic lymph node) Stage IV, and was treated with chemoradiotherapy.

The patient initially received 60 mg/m^2^ docetaxel and 100 mg/m^2^ nedaplatin on day 1, and this was repeated every 3 weeks. The patient experienced an adverse drug reaction, judged to be platinum allergy, after the first treatment, thus the regimen was changed to chemoradiotherapy with S-1 and regional radiation to the primary lung lesion and lymph nodes of #2 L and #7 with a dose of 70 Gy/35. Chest and abdominal CT scan demonstrated a good partial response to chemoradiotherapy in the primary lung lesion and lymph nodes of #2 L and #7. The lymph node of the lesser curvature of the stomach enlarged, and therefore additional radiation was introduced to the enlarged lymph node with a dose of 60 Gy/30. Chest and abdominal CT scan revealed reduction of the primary lung lesion and lymph nodes including lesser curvature of the stomach after this chemoradiotherapy, and no other tumor was detected. Ambulatory follow-up was continued in the department of pulmonary surgery.

**Figure 3:**
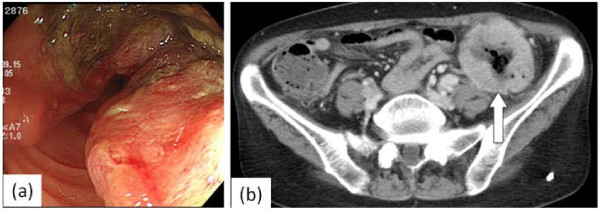
**Colonoscopy discloses bulky disease with stricture in the sigmoid colon.** Abdominal computed tomography scan reveals sigmoid colon tumor invading the abdominal wall unaccompanied by swelling of colonic lymph nodes and distant metastasis.

**Figure 4:**
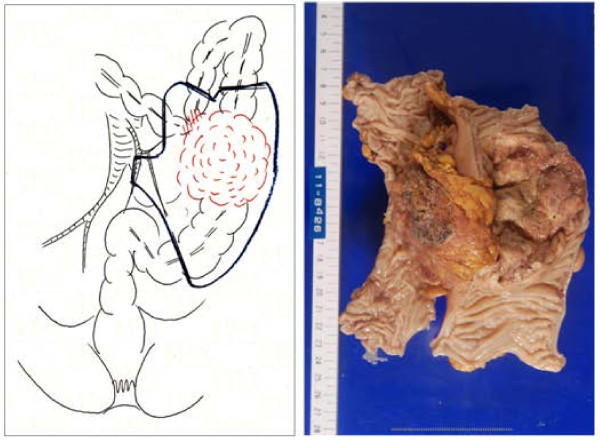
Gross specimen of sigmoid colon shows a bulky tumor invading the transverse colon without nodal involvement.

The patient developed left abdominal pain 6 months later, and colonoscopy disclosed bulky disease with strictures in the sigmoid colon (Figure 3a), diagnosed to be primary colon cancer. The patient was referred to this department. Abdominal CT scan revealed a sigmoid colon tumor invading the abdominal wall, with no swelling of the colonic lymph nodes on distant metastasis (Figure 3b). The sigmoid colon tumor was thought to have rapidly progressed over the months after chemoradiotherapy to the primary lung cancer. She underwent a sigmoid colectomy and partial transverse colectomy for the bulky sigmoid tumor invading the transverse colon for curative resection based on a preoperative diagnosis of primary colon cancer (Figure 4).

**Figure 5:**
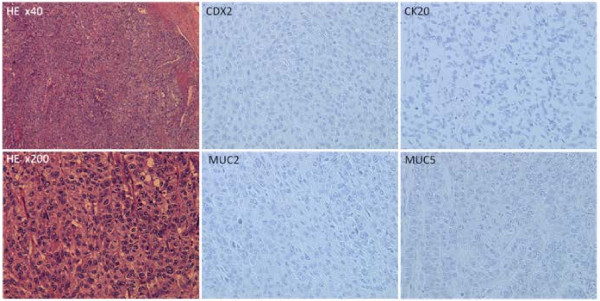
**The pathology specimen demonstrates poorly differentiated squamous cell carcinoma (H&E stain, x40/x200).** The carcinoma cells are negative for CDX2, cytokeratin20, MUC2 and MUC5AC on immunohistochemistry (x200).

The pathology specimen, however, demonstrated poorly differentiated squamous cell carcinoma without metastasis to the colonic lymph nodes, and immunohistochemistry showed that the carcinoma cells were negative for CDX2, cytokeratin20 (CK20), MUC2 and MUC5AC (Figure 5), thus indicating that the carcinoma was not colorectal carcinoma [12-14]. The immunohistological findings suggested the tumor to be metastatic colon cancer from the primary lung carcinoma. Cytology of peritoneal lavage fluid was negative for malignant cells. Her postoperative course was uneventful, and she was discharged 24 days after the operation. She is presently alive at 6 months after the operation, and chemotherapy for the lung cancer was scheduled in the department of pulmonary surgery.

## Discussion

Rossi and colleagues [7] stated that gastrointestinal metastasis from lung cancer has probably been underdiagnosed in living patients because it is frequently regarded as part of a generalized metastatic disease or the lesions are considered to be side effects of chemotherapy, such as ulcers, enteritis, or colitis. Small bowel tumors are likely to present with serious clinical complications such as perforation, obstruction or hemorrhage. Therefore, a number of clinical cases of small bowel metastasis from lung cancer have been reported, while clinical cases of colonic metastases have so far only rarely been reported. Only 11 clinical cases of colonic metastases from lung cancer have been published as case reports [2,10,15-22]. The pathological diagnosis in 10 of the 12 cases, including our presented case, was squamous cell carcinoma. Small cell carcinoma or large cell carcinoma occurred in only one case each.

On the other hand, the most common histological tumor type causing gastrointestinal metastasis varies according across different studies, and every type of lung cancer can result in gastrointestinal metastasis [2,4,6,8,10,11,23,24]. In other words, there is no determinant for any particular cell type to metastasize to the gastrointestinal tract. These data were mostly obtained from the small bowel metastatic cases. More reports of colonic metastasis from primary lung cancer are therefore required to clarify the clinical features.

Regarding the preoperative diagnosis, it is difficult to correctly diagnose the origin of gastrointestinal tumor by CT scan and, even at endoscopy, lung cancer involving the gastrointestinal tract has no peculiar features, mimicking a primary gastrointestinal tumor [7]. Thus, the histological examination is the only way to identify metastatic tumors to the gastrointestinal tract, and immunostaining with TTF-1, CDX2, CK7 and CK20 is also helpful to distinguish primary gastrointestinal carcinoma from metastasis of lung carcinoma [7]. Preoperative diagnosis based on the endoscopic findings was not corrected in the present case. If we had preoperatively performed immunohistochemical examination, correct diagnosis might have been made.

Fecal blood test is useful for early detection of the intestinal metastasis, and is suitable for the first examination for abdominal symptoms [15]. Recently, clinical usefulness of a PET-CT scan is firmly established in primary gastrointestinal carcinoma [2]. Even patients with asymptomatic gastrointestinal metastasis from lung cancer were diagnosed with PET-CT scan in past reports [5,16]. Therefore, PET-CT scan may also play an important role in early diagnosis of colonic metastasis of lung cancer. Gastrointestinal symptoms should be noted in lung cancer patients to avoid underdiagnosis or overlooking colonic metastasis from lung cancer, and to allow early detection with these modalities.

Yang and colleagues [2] reported that the average time from the diagnosis of gastrointestinal metastasis to death was 130 days, indicating poor prognosis. However one report showed a patient remaining alive more than 5 years after resection of metastatic intestine [5]. Although patients with gastrointestinal metastasis from lung cancer are in the latter stages of the disease, early detection and surgical intervention may provide some relief [11].

## Conclusion

This report presented a rare case of colonic metastasis from primary lung cancer. Patients with advanced primary lung cancer that complain of abdominal symptoms may therefore have gastrointestinal metastases from lung cancer, and their gastrointestinal tract should be actively examined to allow early detection and treatment.

## Consent

Written informed consent was obtained from the patient for publication of this Case report and any accompanying images. A copy of the written consent is available for review by the Editor-in-Chief of this journal.

## Abbreviations

CDX2, Caudal-type homeobox 2; CK, Cytokeratin; CT, Computed tomography; Max SUV, Maximum standardized uptake value; PET, Positron emission tomography; TTF, Thyroid transcription factor-1.

## Competing interests

The authors declare that they have no competing interests.

## Authors’ contributions

HS participated in treatment of the patient, contributed to collection of the clinical data and relevant literatures, and to writing of the manuscript. HE participated in treatment of the patient, and helped to edit the manuscript. HT, MT, YK, MS, TA and HO participated in treatment of the patient, and revised and approved the manuscript. KA contributed to histological diagnosis. All authors read and approved the final manuscript.
